# Drug Resistance and Evolvability in an Emerging Human Fungal Pathogen

**DOI:** 10.1128/mbio.01876-22

**Published:** 2022-08-18

**Authors:** R. Blake Billmyre

**Affiliations:** a Stowers Institute for Medical Research, Kansas City, Missouri, USA

**Keywords:** antifungal resistance, *Candida auris*, experimental evolution, hypermutator, subtelomeres

## Abstract

Over the past decade, Candida auris has emerged as a highly transmissible human fungal pathogen. Because of its ability to transmit between patients in hospitals and its ability to rapidly develop drug resistance, C. auris presents unique challenges. However, at a genetic and genomic level we still understand relatively little about how drug resistance develops in this pathogen. Burrack et al. use experimental evolution and whole-genome sequencing to identify mutations correlated with fluconazole resistance in C. auris. They identify interesting genomic features, including highly plastic subtelomeric regions and whole chromosomal and segmental aneuploidies. Excitingly, they also identify the first example of a hypermutator strain in C. auris. In comparison with the model human fungal pathogen Candida albicans, C. auris is more likely to undergo mutation and less likely to undergo copy number variation in response to drug selection, which may be linked to differences in base ploidy level.

## COMMENTARY

Invasive fungal infections are highly difficult to treat because the similarity between humans and fungi has impaired the development of effective and safe antifungal therapeutics. As a result, invasive fungal infections often have extremely high mortality rates (1). In addition, because fungal infections tend to be most prevalent as opportunistic infections of immunocompromised patients, infection frequency has increased dramatically in the past several decades with the development of two very large patient cohorts with chronic immunocompromise: HIV/AIDS individuals and solid organ transplant recipients (2). Both patient cohorts are extremely susceptible to fungal infections, and effective treatment of these diseases is critical to prevent mortality in these populations.

The majority of opportunistic fungal infections over the last several decades have been caused by one of four pathogens, Candida albicans, Cryptococcus neoformans, Aspergillus fumigatus, and Pneumocystis jirovecii, although many other, often regionally restricted, pathogens cause disease as well (1). While there have been outbreaks of related pathogens in new locations like the historically tropical Cryptococcus deuterogattii in the Pacific Northwest of the United States and Canada (3), there have been very few truly novel emergent fungal diseases. However, over the last decade, a previously unknown pathogen called Candida auris has emerged and caused invasive fungal infections across the globe (4, 5).

The emergence of Candida auris as a significant human fungal pathogen has been extremely rapid. C. auris infections appeared roughly simultaneously in multiple locations around the globe (5). Curiously, the emergence of C. auris appears to be best explained by multiple lineages independently gaining virulence potential rather than a single gain of virulence and subsequent distribution around the globe (5). This simultaneous gain of virulence may be caused by climate change (6). Globally distributed C. auris populations may have been exposed to the same selective pressure of elevated temperatures and responded with similar adaptations, resulting in convergent gain of high temperature growth. Because the ability to grow at human body temperature is a key virulence factor, this change may have resulted in a gain of virulence potential in humans. If so, C. auris may be the first in a wave of emerging fungal diseases generated by this same selective pressure (7).

C. auris is particularly dangerous because it is capable of effective patient-to-patient transmission within health care settings (8), unlike many other common invasive fungal pathogens. C. neoformans infections, for example, are environmentally acquired, and the human host is thought to be a dead end, with no return to the environment or patient-to-patient transmission (9). Thus, while drug resistance that emerges during the treatment of one cryptococcosis patient is dangerous for that patient, it is highly unlikely to result in infection of subsequent individuals. Instead, drug resistance must generally develop *de novo* in every patient, although environmental exposure to antifungals may be selecting for drug resistance in environmental reservoirs of pathogens like Aspergillus prior to human infection (10, 11). However, because C. auris can transmit between patients, drug resistance and multidrug resistance is even more dangerous. Over time, treatment and transmission can quickly result in emergence of multidrug resistant pathogens, as it commonly does in bacterial pathogens. In fact, multidrug resistance is increasingly common in C. auris and presents substantial clinical challenges (12).

However, we have comparatively little understanding of the genetic mechanisms underpinning drug resistance in C. auris, in part because it emerged as a pathogen so recently. In a recent study, Burrack et al. used experimental evolution to explore how drug resistance emerges *in vitro* in clinically derived strains of C. auris (13). The authors passaged 17 independent clinical isolates in both high and low doses of fluconazole, a representative drug from one of the five primary classes of antifungal agents (azoles, candins, polyenes, 5FC, and the newly approved ibrexafungerp). Because fluconazole and other azole class drugs are commonly used to treat many invasive fungal diseases, we have a reasonable understanding of the spectrum of potential drug resistance mutations in several other pathogens, but not in C. auris.

Fluconazole, like other azole drugs, acts by inhibiting the function of Erg11, a critical component in the ergosterol biosynthesis pathway. *ERG11* is an essential gene in fungi but lacks an ortholog in humans, where cholesterol rather than ergosterol is the primary membrane sterol. As a result, inhibiting Erg11 is a highly effective mechanism for treating fungal infections. Resistance to fluconazole typically takes place through a few known pathways that either alter binding of fluconazole to Erg11, decrease the amount of fluconazole in the cell, or increase the amount of Erg11 in the cell (14). Alterations in Erg11 sequence can decrease fluconazole binding and reduce inhibition. Mutations that cause reduction in drug import or increase drug export can reduce the amount of fluconazole in a cell, thereby decreasing the effective dose the fungal cell experiences during treatment. Finally, increases in the amount of Erg11, through either overexpression of Erg11 or increases in *ERG11* gene copy via aneuploidy, can increase the ratio of Erg11 to fluconazole and restore Erg11 activity even at dosages that would typically be inhibitory.

Burrack et al. showed that fluconazole resistance in C. auris can develop extremely rapidly *in vitro*. Over only 9 days of growth, two of their selected lines doubled their IC_50_ (inhibitory concentration 50), the drug concentration at which growth is decreased by 50%. Further, clinical strains with existing resistance were stable even in the absence of drug, suggesting that resistance may develop more rapidly than it is lost. They also subjected their evolved lines to whole-genome sequencing to determine the spectrum of mutations that occurred under fluconazole selection alongside sequencing of the original clinical lines. One highlight of their findings was rampant variation within the subtelomeric regions of these clinical isolates, as the authors note that all 17 clinical isolates they assayed had at least one subtelomeric deletion relative to the reference genome. Further, individual colonies derived from the evolved lines contained additional subtelomeric deletions, suggesting that this process is rapid and ongoing. Subtelomeric variation is common in eukaryotic microbes and may be a driver of phenotypic diversity and adaptability during infection (15). For example, variation in subtelomeric content is critical for trypanosomes to generate antigenic variation during infection (16). In C. auris, subtelomeres are enriched for transporters, suggesting that variation could affect the ability to acquire critical nutrients during infection. Future experiments exploring the impact of subtelomeric variability in C. auris will likely be highly informative.

In addition, Burrack et al. identified point mutants and aneuploidies linked to proposed resistance loci in C. auris (13). They identified point mutations in *ERG11*, the target of fluconazole, in *TAC1B*, a regulator of drug efflux pumps, and in *UPC2*, a regulator of ergosterol biosynthesis genes. While mutations in *ERG11* and *TAC1B* have previously been correlated with resistance in C. auris (5, 17–19), mutations of *UPC2* have not, but are known to confer resistance in C. albicans (20). In addition to the point mutations observed, one evolved line was aneuploid for the chromosome containing *TAC1B* as well, and one individual colony sequenced from another line was aneuploid for the chromosome containing *ERG11*. Notably, they also identified resistant isolates lacking mutations in known resistance genes. One exciting novel resistance candidate identified was a mutation in *DCR1*, a canonical component of the RNAi pathway. This suggests the possibility for RNAi-mediated gene regulation driving drug resistance and sensitivity in C. auris. RNAi can drive drug resistance through epimutation in another fungal pathogen, Mucor circinelloides (21, 22). As the genetic tools for C. auris continue to improve (23), exploring these candidates will reveal interesting aspects of drug resistance in C. auris.

Finally, the authors identified a hypermutator isolate with a mutation in the C. auris ortholog of *MLH1*, resulting in approximately a 10-fold increase in mutation rate relative to the other wild-type isolates. Mlh1 is important for mismatch repair. Inactivation causes dramatically elevated mutation rates, particularly in simple repeats such as homopolymer runs, and can cause hereditary colon cancer in humans (24). For fungal pathogens, this identification is part of a wave of recently identified hypermutator isolates identified in multiple fungal pathogens, most notably starting in Candida glabrata, where mismatch repair defective strains can make up extremely high proportions of the isolates (25–27). Subsequently, hypermutators have been identified in C. neoformans and Cryptococcus deuterogattii as well (28–30). Multiple studies have demonstrated that hypermutator isolates can develop resistance to antifungal agents more rapidly than nonhypermutator isolates.

Notably, all of the fungal pathogens where hypermutators have been observed, including C. auris, are haploids. In contrast, despite robust studies of drug resistance in the diploid C. albicans, no hypermutator isolates have been described. This may be true for two reasons. First, it may be easier to generate hypermutators in a haploid than in a diploid. A single loss of function mutation in a DNA repair gene from a haploid produces a hypermutator, while in a diploid that mutation will likely be masked by the remaining wild-type allele. Second, hypermutators may be more advantageous in a haploid than a diploid. As the authors point out, the ability of mutation to drive phenotypic change is damped in a diploid like C. albicans because the wild-type allele will frequently mask the effects of a single loss of function allele. Losing gene function in C. albicans typically requires a two-step process involving either independent mutations in both alleles of a gene or a mutation of one allele followed by a loss of heterozygosity event. Instead of point mutations, resistance in C. albicans is more frequently driven by copy number variation (31), including aneuploidy, which appears to be less common in C. auris. The phenotypic consequences of aneuploidy are different in haploids and diploids. For example, gain of 1 chromosome aneuploidy changes dosage of the genes located on that chromosome by only 50% in a diploid, while in a haploid the same gain of 1 chromosome aneuploidy changes dosage of those genes by 100%. Diploids experience both damped positive effects (drug resistance) and damped negative effects (perturbations in protein homeostasis) from aneuploidy, relative to haploids.

This study provides an excellent entry to experimental evolution in an important emerging human fungal pathogen. The similarities and contrasts with existing studies of drug resistance in C. albicans will provide fodder for numerous fruitful explorations of drug resistance, both in understanding the specific details of resistance in C. auris and in establishing general principles that underlie the differences between evolvability in these pathogens.
